# Motivation towards medical career choice and future career plans of Polish medical students

**DOI:** 10.1007/s10459-014-9560-2

**Published:** 2014-10-29

**Authors:** Jakub Gąsiorowski, Elżbieta Rudowicz, Krzysztof Safranow

**Affiliations:** 1 Medical Education Unit, Pomeranian Medical University, ul. Grudziądzka 31, 70-103 Szczecin, Poland; 2 Szczecin, Poland; 3 Department of Biochemistry and Medical Chemistry, Pomeranian Medical University, Szczecin, Poland

**Keywords:** Career expectations, Career motivation, Medical students, Polish, Professional plans

## Abstract

This longitudinal study aimed at investigating Polish medical students’ career choice motivation, factors influencing specialty choices, professional plans and expectations. The same cohort of students responded to the same questionnaire, at the end of Year 1 and Year 6. The Chi-square, Mann–Whitney U tests and logistic regression were used in analyses. The results showed that altruistic and scientific reasons were the main motives for choosing a medical career. The motives remained stable over time. The effect of gender on altruistic motivation was stronger at the end of the study, with females’ rating higher. The most favored career paths were associated with non-primary care specializations and work in a hospital. Results of the multivariate logistic regression showed that primary care specializations were more attractive to females, final year students, those from small agglomerations, and those less concerned about high earnings. Preferences regarding sector of work were formed at later stages of training. A preference shift was observed, between Year 1 and Year 6, towards favoring work in the public sector. Predictors of the desire to work in the public sector were: being a male and the final year student, paying less attention to high earnings, wanting certainty of finding work, having a stronger need for interesting and socially important work. A significant decline in the level of interest in seeking employment abroad was observed with the progress of studies. Our findings are likely to provide useful information for educators, policy planners and policy makers.

## Introduction

Political, economic and social changes in former East European countries in the last two decades have had an enormous impact on many areas of life, including people’s values, aspirations and work attitudes. The accession of Poland to the European Union (EU) in 2004 and the European Council Directive 2005/36/EEC, allowing health professionals full registration in any EU state, created new work opportunities for Polish medical doctors. Between 2004 and 2007, more than 6,000 physicians successfully applied for the professional verification certificate, allowing them to apply for work in other EU countries. In the same period of time 2,961 Polish doctors were newly registered to practice in EU countries. The majority of them were specialists in anesthesiology, internal medicine and general surgery (Leśniowska 2007). In addition, Polish Medical Schools started to offer English-language Programs aimed at international students. In 2012 more than 6,000 foreign students were studying medicine or dentistry in Poland. The majority of them came from Scandinavian countries and the USA (online report: study in Poland 2012). These changes brought new challenges to medical educators, health care planners and managers, both locally and internationally. The initial challenge to the medical faculty was to develop their skills to enable teaching in English for students coming from different linguistic and socio-cultural backgrounds. These changes resulted in wider use of the English language at universities and enrichment of academic life for Polish students. Although, there is no hard data, it is quite likely that the internationalization of university life would make seeking employment or further studies abroad a more realistic option for Polish medical students.

In that context, studies into students’ motivation for choosing medicine as a profession and their plans related to becoming a physician seem to be of prime importance to policy makers and educators (Gibis et al. 2012). Data from previous investigations show that medical students’ motives, beliefs, and values around their studies and future profession have an impact on the eagerness to accept the training program and on learning and academic performance as well as on the choices made for future career paths. The congruence between students’ priorities regarding medicine and those of the university at which they receive their education is central for achieving pedagogical goals (Draper and Louw 2007; Khader et al. 2008).

Studies of motivation towards career choice of medical students have been carried out among applicants to medical school (McManus et al. 2006), freshmen (Millan et al. 2005; Baboolal and Hutchinson 2007; Draper and Louw 2007), final year students/graduates (Dastjerdi et al. 2012; de Vries et al. 2010) students in selected years (Aasland et al. 2008; Avgerinos et al. 2006; Amin et al. 2009; Crossley and Mubarik 2002; Diderichsen et al. 2011; Drinkwater et al. 2008; Puljak et al. 2007), as well as all students registered at an institution (Al-Fouzan et al. 2012; Ferrinho et al. 2011; Heiligers 2012; Mandeville et al. 2012). The results of these studies are often inconsistent. Some researchers reported a prevalence of humanitarian motives, e.g., ‘opportunity to help and care for people’, ‘making a difference’, ‘humanity of medicine’ and scientific reasons, e.g. ‘interest in human body structure and function’, ‘interest in science’, ‘opportunity to perform research’ (Puljak et al. 2007; Draper and Louw 2007; Crossley and Mubarik 2002), whereas others revealed the important role of such motives as desire to have power, control, knowledge (McManus et al. 2006), and social status (McManus et al. 2006; Dastjerdi et al. 2012). It remains unclear if humanitarian and scientific motives to study medicine are stable during the course of study (Draper and Louw 2007) or if they decrease significantly (Puljak et al. 2007). Exploratory surveys among Polish students revealed that the reasons most often indicated for choosing medical studies were internal motives such as ‘a wish to help others’ and ‘an interest in medical subjects and issues’. However, the frequency of these types of motivators was more prevalent among year one students than among graduating ones (Gąsiorowski et al. 2008, 2010).

Another area of interest to health educators, health services planners and developers are studies aiming at the identification of factors determining medical career preferences and choices. Studies reveal that future career plans connected with specialty choices depend on numerous factors, which can be broadly divided into a number of categories including demographics, medical school ethos and curriculum, students’ perceptions of specialty characteristics and student-held attitudes and values (Newton et al. 1998). Among demographic factors, gender (Buddeberg-Fischer et al. 2006; Khader et al. 2008; Heiligers 2012; de Vries et al. 2010; Fukuda and Harada 2010), age (Kusurkar et al. 2010), rural or city origin (de Vries et al. 2010), marital status (Heiligers 2012; Newton et al. 1998), and parents’ education (Dastjerdi et al. 2012) were shown as having some association with the choice of specialty. Amid these demographic factors, gender seems to have the most influence on specialty choice. Perceiving specialty as intellectually challenging (Avgerinos et al. 2006; Al-Fouzan et al. 2012; Harris et al. 2005; Khater-Menassa and Major 2005; de Vries et al. 2010), or giving opportunity for the direct interaction with patients (Avgerinos et al. 2006; Vaglum et al. 1999; Knox et al. 2008), or leading to good treatment outcomes (Al-Fouzan et al. 2012; Baboolal and Hutchinson 2007), seem to be the major factors influencing specialty preferences.

It has been noted that students preferences for specialty choice, as well as their perceptions of motivation for medical career choice, have a tendency to change during the course of their studies (Compton et al. 2008). However, there is a scarcity of longitudinal studies allowing the collection of important data for our understanding of the dynamics and direction of changes within the same group of students at different points during their studies. Thus, the aim of our longitudinal study presented in this paper was to investigate Polish medical students perceived motivation for choosing medicine as a career and examine their career expectations, specialty choices and career plans in relation to such demographics as gender, age, year of study, place of origin, and parents’ education.

## Methods

### Participants

This survey was carried out among medical students at the Pomeranian Medical University (PMU), Poland. Participants were tested with the same set of questions twice. The first time at the end of Year 1 (n = 143 of 166 students registered in 2003/2004) and the second time at the end of Year 6 (n = 119 of 135 students registered in 2008/2009), just before the graduation in 2009. The attrition rate was 18.6 % over a 5 year period. In spite of attrition, the cohort’s demographic characteristics, at two points of data collection remained comparable. The proportion of female to male students remained at the level 1.5:1. The mean age was 20.3 years (*SD* = 0.8) for Year 1 students and 25.5 years (*SD* = 0.9) for Year 6 students.

Students entered Medical School after 12 years of education (which in Poland starts at the age of 7) ending in matriculation exam. Results of matriculation exam decide about students admission to university. The acceptance rate to the PUM medical program is around 30 %. The 6-year graduate curriculum at the PMU is comparable to those in other Polish medical schools.

### Instrument

A piloted questionnaire was developed by the first author, for an extensive study aiming at measuring the PMU’s students perceptions of different aspects of studying at medical school. The questionnaire was anonymous. The data presented in this article are based on students’ responses to seven categorical questions with multiple-choice responses. Demographic background information about participants’ age, gender, parents’ education was also collected.

Participation in the survey was voluntary and was carried out at the end of a seminar. All students agreed to fill in the questionnaire, despite the lack of incentives. Students were assured about anonymity. In the case of this study the ethical approval was not required.

To explore reasons for studying medicine as perceived by students, the following question, requiring ranking the study reasons, was used:

What made you decide to study at the Faculty of Medicine? (This is a multiple choice question. Please rank the selected responses by writing numbers in the grid: 5—the most important reason; 4—the second important reason; 3—the third important reason, and so on.)

- An interest in medical subjects and medical issues
- The desire to help others as medical doctor in the future
- The desire to obtain a profession with high social prestige
- The desire to obtain a well-paid profession in the future
- The desire to continue the family tradition
- Other reason/s (What were they?)

Students’ expectations related to being a medical doctor, as perceived at the beginning of the study (end of Year 1) and just before the graduation were surveyed with the use of following question:

What are your expectations related to being part of the medical profession in the future? (This is a multiple choice question. Please rank the selected responses by writing numbers in the grid: 5—the most important expectation; 4—the second important expectation; 3—the third important expectation, and so on.)

- The prospect of performing socially important and interesting work
- The certainty of finding work
- The possibility of achieving high earnings
- Gaining high social status and prestige
- The possibility of further career development
- Other expectation/s (what are they?)

In the above questions students didn’t have to rank all responses.

The following five questions were aiming at obtaining information regarding students’ career plans and career choices, as they perceived them at the beginning and at the end of medical studies.

1. In the future, which sector would you like to work in?
  Public sector
  Private sector
  I do not know yet

2. In the future, which setting you would like to work in?
  Hospital
  Outpatient clinic
  Private clinic
  Hospice
  Other setting (specify)
  I do not know yet

3. In the future, what capacity you would like to work in?
  As a family doctor
  As a specialist (please give the name of specialization)
  I do not know yet

4. Are you considering the possibility of research work related to medicine?
  NO YES (Please indicate in which field……………………)

5. Where would you like to work as a doctor in the future?
  In Poland
  Outside Poland (e.g. in the European Union)
  I do not know yet

### Study design and data analysis

The same cohort of students was surveyed in a group setting with the same set of written questions at two different points in their studies, at the end of year one and just before the graduation. This longitudinal approach allowed us to explore potential changes in students’ career expectations and career plans over time.

The collected data were entered into a database and analyzed using the Statistica 10 Packet (StatSoft). A *p* value ≤.05 was set as a criterion for establishing statistical significance.

Group differences were analyzed using the Chi-square test and the Mann–Whitney U test. This nonparametric test was employed because the assumptions for using independent samples *t* tests were not upheld. Furthermore, multivariate logistic regression analyses were conducted to determine independent predictors of three binary dependent variables: (a) preferred specialization: primary care (PC) defined as family medicine, general internal medicine and general pediatrics versus non-primary care (non-PC),defined as all other specializations (Newton et al. 1998); (b) work migration plans: career abroad versus career in Poland; (c) preferred sector of work: private versus public. The independent variables entered into the model consisted of two broad categories of predictors. The first category contained three demographic factors, namely, gender, year of study, and place of origin. The second category, included five expectations related to being a doctor, that is, performing socially important and interesting work, certainty of finding work, possibility of achieving high earnings, having high social status and prestige, and further career development. We included those variables in the regression model because they were found to be significant in our initial univariate analyses or in previous studies.

## Results

A total of 143 questionnaires were completed by Year 1 students and 119 by Year 6 students. This represented a response rate of 86.1 and 88.1 % respectively, based on the total of 166 students registered in Year 1 and 135 in Year 6.

Nearly half of respondents originated from cities with populations >100,000 (n Year 1 = 71; n Year 6 = 62). Around 35 % (n Year 1 = 49; n Year 6 = 38) came from cities of <100,000 inhabitants, and around 15 % (n Year 1 = 23; n Year 6 = 19) originated from villages.

The majority of students’ parents completed tertiary education. The respective percentages for fathers and mothers of Year 1 students were 65.4 % (n = 93) and 64.1 % (n = 91), whereas for Year 6 students were 64.7 % (n = 77) and 67.2 % (n = 80). Of these parents, the percentages that had medical degree (physician, dentist, pharmacist or medical analyst) were 16.2 % (n = 23) for fathers and 19 % (n = 27) for mothers of Year 1 students and 16.8 % (n = 20) for fathers and 17.6 % (n = 21) for mothers of Year 6 students. Comparison of means regarding parental education of male and female students showed that male students had higher educated fathers than female students. The difference was statistically significant for Y1 (U = 1,767, z = − 2.95, *p* < .01) and close to statistical significance for Y6 (U = 1,374.5, z = − 1,85, *p* = .06).

The observed variations between Year 1 and Year 6 students in regard to the figures related to the ‘place of origin’ and ‘parents education’ were due to attrition over 5 years of study. However, none of the differences were statistically significant.

Looking at reasons for undertaking medical studies, the two most highly rated reasons were ‘the desire to help others’ (rated 1st in Year 1 and 2nd in Year 6) and ‘an interest in medical subjects and medical issues’ (rated 2nd in Year 1 and 1st in Year 6). ‘The desire to obtain a profession with high social prestige’ was in third place, followed by ‘the desire to obtain a well-paid profession’. A comparison of Year 1 and Year 6’s ratings showed a statistically significant difference between the groups with respect to one category only, namely, ‘the desire to help others’ (U = 5,138, z = 2.54, *p* < .02, Cohen’s d value of 0.346). The observed group difference was due to a drop in the level of ratings by male respondents. The difference in males ratings in Year 1 and Year 6 was statistically significant (U = 529.5, z = 3.63, *p* < .001). The observed effect size was large (Cohen’s d value 0.852). Females ratings in the category ‘the desire to help’ remained relatively stable throughout the studies. Thus, in Year 6 females ratings in that category were significantly higher than males ratings (U = 756.5, z = 2.87, *p* < .01, Cohen’s d = 0.549). The detailed results are shown in Table 1.

**Table 1:** Reasons to undertake medical study by year of study and gender: means (X), standard deviations (SD), male (M), female (F) and total (T)

Reason (category)	Mean importance rating		
	M	F	T
	X (SD)	X (SD)	X (SD)
An interest in medical subjects and issues			
Year 1 (M = 50, F = 74, T = 124)	4.20 (1.05)	4.26 (0.84)	4,23 (0.93)
Year 6 (M = 39, F = 67, T = 106)	4.36 (0.84)	4.13 (1.11)	4,34 (0.81)
The desire to help others as a doctor in the future			
Year 1 (M = 49, F = 79, T = 128)	4.61 (0.67)^b^	4.48 (0.86)	4,53 (0.79)^a^
Year 6 (M = 37, F = 60, T = 97)	3.89 (0.99)^b,c^	4.42 (0.94)^c^	4,22 (0.99)^a^
The desire to obtain a profession with high social prestige			
Year 1 (M = 41, F = 62, T = 103)	3.41 (0.95)	3.42 (0.92)	3.42 (0.92)
Year 6 (M = 34, F = 41, T = 75)	3.56 (0.99)	3.41 (0.74)	3.48 (0.86)
The desire to obtain a well-paid profession in the future			
Year 1 (M = 37, F = 47, T = 84)	3.27 (1.15)	3.21 (1.14)	3.24 (1.14)
Year 6 (M = 27, F = 27, T = 54)	3.19 (1.30)	3.04 (1.06)	3.11 (1.18)
The desire to continue the family tradition			
Year 1 (M = 18, F = 15, T = 33)	3.28 (1.41)	2.93 (1.53)	3.12 (1.45)
Year 6 (M = 8, F = 15, T = 23)	3.63 (1.19)^d^	2.20 (1.08)^d^	2,70 (1.29)

^a^ *p* < .02, Cohen’s d = 0.346

^b^ *p* < .001, Cohen’s d = 0.852

^c^ *p* < .01, Cohen’s d = 0.549

^d^ *p* < .01, Cohen’s d = 1.258

When asked to rank their expectations related to being part of the medical profession, all students assigned the highest rank to ‘the prospect of performing socially important and interesting work’ followed by ‘the certainty of finding work’, and ‘the possibility of further career development’. The lowest rank was assigned to ‘the possibility of achieving high earnings’ and the second lowest was given to the expectation of ‘gaining high social status and prestige’. A comparison of Year 1 and Year 6 students’ total rating scores for different expectation categories showed no statistically significant differences.

With respect to the gender comparisons, no gender differences were observed in professional expectations among Year 1 students. Among Year 6 students one such difference was noticed, namely females ranked the expectation ‘to perform socially important and interesting work’ higher than males (U = 989, z = 2.17, *p* < .05, Cohen’s d = 0.472). Looking at differences over time (Year 1 and Year 6) for each gender separately, some differences were found, although they were not statistically significant. Male students at Year 6 ranked ‘the certainty of finding work’ and ‘gaining high social status and prestige’ lower than at the beginning of their medical studies. Among female students, a slump in expectations between Year 1 and Year 6 was observed in two areas ‘gaining high social status and prestige’ and ‘the possibility of further career development’. Table 2 contains detailed results.

**Table 2:** Expectations related to becoming a part of medical profession by year of study and gender: means (X), standard deviations (SD), males (M), females (F) and total (T)

Expectation (category)	Mean importance rating		
	M	F	T
	X (SD)	X (SD)	X (SD
The prospect to perform socially important and interesting work			
Year 1 (M = 54, F = 79, T = 133)	4.59 (0.81)	4.70 (0.65)	4.65 (0.72)
Year 6 (M = 40, F = 62, T = 102)	4.35 (0.98)^a^	4.73 (0.58)^a^	4.58 (0.78)
The certainty of finding work			
Year 1 (M = 32, F = 48, T = 80)	3.47 (1.29)	3.56 (1.25)	3.53 (1.26)
Year 6 (M = 31, F = 50, T = 81)	3.87 (1.06)	3.84 (1.00)	3.85 (1.01)
The possibility of further career development			
Year 1 (M = 40, F = 59, T = 99)	3.40 (1.28)	3.69 (0.93)	3.58 (1.09)
Year 6 (M = 34, F = 45, T = 79)	3.32 (1.20)	3.33 (1.31)	3.33 (1.26)
Gaining high social status and prestige			
Year 1 (M = 35, F = 53, T = 88)	3.46 (0.92)	3.15 (1.15)	3.27 (1.07)^b^
Year 6 (M = 32, F = 34, T = 66)	3.09 (1.30)	2.79 (1.23)	2.94 (1.26)^b^
The possibility of achieving high earnings			
Year 1 (M = 37, F = 44, T = 81)	3.05 (1.20)	2.91 (1.36)	2.98 (1.28)
Year 6 (M = 34, F = 38, T = 72)	2.91 (1.26)	2.97 (1.22)	2.94 (1.23)

^a^ *p* < .05, Cohen’s d = 0.472

^b^ *p* = .08

Data about students’ professional plans consisted of their responses to five questions concerning: preferred sector and setting of work, planned choice of specialization, consideration of a research career, and desire to work abroad. With regard to preferred sector of work, more than 40 % (n = 57) of Year 1 students did not know if they would prefer to work in the private or public sector. In contrast, all Year 6 students indicated their preferences. Of those Year 1 students who specified their preferences, almost two-thirds (n = 47, 58 %) favored work in private sector, almost one-third (n = 24, 29.6 %) in public sector and 12 % (n = 10) wanted to combine work across both sectors. In contrast, Year 6 students favored work in public sector (n = 46, 40 %) or combination of public and private sector work (n = 43, 37.4 %) and only a minority preferred work in the private sector. The differences between Year 1 and Year 6 students were statistically significant regarding preference for public sector (χ^2^ = 16.02, *df* = 1, *p* < .001) and for combined private and public sector (χ^2^ = 34.42, *df* = 1, *p* < .001).

When asked about a preferred work setting, most students had clear preferences. The percentage of undecided students in Year 1 and Year 6 was rather low and comparable (around 15 %). The majority of respondents, in both years, pointed to a hospital as being their preferred work setting, followed by private clinic. Hence, when asked about favoring career either in a primary care (PC) field or a non-primary care (non-PC) fields about 50 % of all Year 1 and Year 6 students chose non-PC. This percentage increased to 77 % in Year 1 and 67 % in Year 6, when undecided responses were excluded. The number of ‘I do not know’ responses decreased with the progress of studies (χ^2^ = 6 .95, *df* = 1, *p* < .05), whereas the number of students favoring a PC career increased (χ^2^ = 4 .62, *df* = 1, *p* < .05). Future involvement in research work proved to be attractive to about half of the respondents, irrespective of gender or year of study.

To test the possible impact of parental medical education on student’s specialty choice, we have compared preferences of two groups of final year students. One group had at least one parent with a medical degree (n = 29), whereas the other group had parents with a non-medical education. Preferences of students belonging to the former group were as follows: 67.8 % (n = 19) showed interest in a non-PC specialty, 17.9 % (n = 5) in a PC specialty and 14.3 % (n = 4) marked ‘don’t know’ and one respondent didn’t provide any response. The results of students belonging to the latter group were: 49. 4 % (n = 44) chose a non-PC specialty, 28.1 % (n = 25) a PC specialty, 22.5 % (n = 20) marked ‘don’t know’ as their response and one respondent didn’t provide any response. The observed differences between the two groups in regard to declared preferences of specialty and undecided tested with χ^2^ (*df* = 2) was not statistically significant (*p* = 0.23).

The proportion of respondents declaring Poland as the place of their future medical career increased from about one-third in Year 1 to above half in Year 6 (χ^2^ = 11.65, *df* = 1, *p* < .001). At the same time, the percentage of those who planned work migration decreased by half (χ^2^ = 7.80, *df* = 1, *p* < .01). About one-third of respondents was uncertain about their preferred country of work, and no variations were observed here across gender or year of study. The detailed results are shown in Table 3.

**Table 3:** Professional plans by year of study and gender: males (M), females (F) and total (T)

Professional plans	Year 1			Year 6		
	F (n = 86)	M (n = 57)	T (n = 143)	F (n = 72)	M (n = 47)	T (n = 119)
	n (%)	n (%)	n (%)	n (%)	n (%)	n (%)
Sector of work						
Public	12 (14.6)	12 (21.4)	24 (17.4)^a^	25 (35.7)	21 (46.7)	46 (40.0)^a^
Private	30 (36.6)	17 (30.4)	47 (34.1)	16 (22.9)	10 (22.2)	26 (22.6)
Public and private	7 (8.5)	3 (5.4)	10 (7.2)^b^	29 (41.4)	14 (31.1)	43 (37.4)^b^
Do not know	33 (40.3)	24 (42.8)	57 (41.3)	–	–	–
No answer	4	1	5	2	2	4
Setting of work						
Hospital	39 (47.6)	29 (51.8)	68 (49.2)	39 (55.7)	32 (72.7)	71 (62.3)
Private practice	19 (23.2)	10 (17.9)	29 (21.0)	13 (18.6)	8 (18.2)	21 (18.4)
Outpatient clinic	2 (2.4)	2 (3.6)	4 (2.9)	2 (2.9)	–	2 (1.7)
Combined settings	4 (4.9)	4 (7.1)	8 (5.8)	–	–	–
Hospice and other places	5 (6.1)	2 (3.6)	7 (5.0)	–	1 (2.3)	1 (0.9)
Do not know	12 (14.6)	9 (16.1)	21 (15.1)	16 (22.9)	3 (6.8)	19 (16.7)
No answer	4	1	5	2	3	5
Specialization						
PC	15 (18.3)	5 (9.1)	20 (14.6)^c^	20 (28.2)	10 (21.7)	30 (25.7)^c^
nPC	37 (45.1)	30 (54.5)	67 (48.9)	32 (45.1)^g^	31 (67.4)^g^	63 (53.8)
Do not know	30 (36.6)	20 (36.4)	50 (36.5)^d^	19 (26.7)^h^	5 (10.9)^h^	24 (20.5)^d^
No answer	4	2	6	1	1	2
Research work						
Yes	38 (48.1)	33 (60.0)	71 (53.0)	33 (47.8)	22 (47.8)	55 (47.8)
No	41 (51.9)	22 (40.0)	63 (47.0)	36 (52.2)	24 (52.2)	60 (52.2)
No answer	7	2	9	3	1	4
Migration plans						
Poland	28 (34.6)	20 (35.8)	48 (35.0)^e^	44 (62.0)	22 (47.8)	66 (56.4)^e^
Other country	22 (27.1)	18 (32.1)	40 (29.2)^f^	7 (9.9)	10 (21.7)	17 (14.5)^f^
Do not know	31 (38.3)	18 (32.1)	49 (35.8)	20 (28.1)	14 (30.5)	34 (29.1)
No answer	5	1	6	1	1	2

^a^χ^2^ = 16.02 (*df* = 1), *p* < .001

^b^χ^2^ = 34.42 (*df* = 1), *p* < .001

^c^χ^2^ = 4.62 (*df* = 1), *p* < .05

^d^χ^2^ = 6.95 (*df* = 1), *p* < .05

^e^χ^2^ = 11.65 (*df* = 1), *p* < .001

^f^χ^2^ = 7.80 (*df* = 1), *p* < .01

^g^χ^2^ = 5.60 (*df* = 1), *p* < .05

^h^χ^2^ = 4.32 (*df* = 1), *p* = .059

The results of logistic regression analysis (Table 4) in which the ‘preferred specialization’ was entered as the dichotomous dependent variable showed that the statistically significant predictors of primary care (PC) career choice, were three demographic variables: coming from a city of <100,000 inhabitants (*p* < .001), being a sixth year student (*p* < .02) and being a female (*p* < .05), and a single variable from the category ‘expectations related to being a doctor’, i.e., attaching low importance to high earnings (*p* < .05).

**Table 4:** Logistic regression models for predictors of primary care career, further career in Poland and sector of work: odds ratio (OR), 95 % CI and p values

Dependent variable	Independent variables	OR (95 % CI)
Specialization PC versus nPC	Socio-demographic factors	
	Gender (female vs male)^d^	2.33 (1.04; 5.00)
	Year of study (I vs VI)^c^	0.38 (0.17; 0.83)
	Place of origin (city <100,000 vs city >100,000 inhabitans)^a^	3.57 (1.69; 7.69)
	Expectations related to become a doctor	
	The prospect of performing socially important and interesting work	1.23 (0.90; 1.67)
	The certainty of finding work	0.85 (0.69; 1.05)
	The possibility of achieving high earnings^d^	0.75 (0.57; 0.97)
	Gaining high social status and prestige	1.20 (0.95; 1.54)
	The possibility of further career development	0.98 (0.80; 1.20)
Migration plans Poland versus Abroad	Socio-demographic factors	
	Gender (female vs male)	1.52 (0.74; 3.03)
	Year of study (I vs VI)^a^	0.28 (0.13; 0.59)
	Place of origin (city <100,000 vs city >100,000 inhabitants)	1.19 (0.59; 2.38)
	Expectations related to become a doctor	
	The prospect of performing socially important and interesting work	0.97 (0.74; 1.27)
	The certainty of finding work	1.08 (0.88; 1.30)
	The possibility of achieving high earnings^c^	0.75 (0.60; 0.95)
	Gaining high social status and prestige^e^	1.22 (0.99; 1.52)
	The possibility of further career development	0.90 (0.73; 1.10)
Sector of work Public versus private	Socio-demographic factors	
	Gender (female vs male)^d^	0.41 (0.17; 1.00)
	Year of study (I vs VI)^a^	0.13 (0.05; 0.34)
	Place of origin (city <100,000 vs city >100,000 inhabit.)^e^	0.46 (0.20; 1.05)
	Expectations related to become a doctor	
	The prospect of performing socially important and interesting work^c^	1.52 (1.06; 2.13)
	The certainty of finding work^b^	0.68 (0.53; 0.88)
	The possibility of achieving high earnings^b^	0.65 (0.50; 0.86)
	Gaining high social status and prestige	0.83 (0.65; 1.08)
	The possibility of further career development	0.89 (0.69; 1.15)

^a^ *p* < .001; ^b^ *p* < .01; ^c^ *p* = .02; ^d^ *p* < .05; ^e^ *p* = .06

The logistic regression model in which the binary dependent variable was ‘preferred work sector’ showed that the strongest demographic predictor of choosing public sector of work was being a sixth year student (*p* < .001) followed by being a male (*p* < .05), whereas coming from a city of more than 100,000 inhabitants almost reached the statistical significance (*p* = .06). From the category ‘expectations related to being a doctor’ three variables proved to be the statistically significant predictors of preference for work in public sector. The two strongest were attaching low importance to ‘the certainty of finding work’ and to ‘the possibility of achieving high earnings’ (*p* < .01). The third predictor was attaching importance to ‘the prospect of performing socially important and interesting work’ (*p* = .02).

When predictors of ‘work migration plans’ were analyzed using the logistic regression model two predictors of ‘choosing Poland as a preferred place of medical career’ reached the statistically significant level. Of these predictors, one was from the group of demographic variables, ‘being a sixth year student’ (*p* < .001) and the other one was from the ‘expectations related to being a doctor’ category, i.e. attaching low importance to high earnings. Whereas, attaching importance to ‘gaining high social status and prestige’ almost reached statistical significance (*p* = .06).

## Discussion

This longitudinal investigation provided empirical data regarding motivation for studying medicine, future career expectations, specialty choices and career plans, from a sample of Polish medical students. The influence of demographic and socio-economic factors on the examined variables was also considered.

The attrition rate of 18.6 % during the 5 years of studies seems high, but it includes students who transferred to other medical courses, took advantage of the dean’s leave, postponed their graduation to next year(s) or gave up studies due to academic or personal reasons. Our results regarding ‘the total attrition rate’ are similar (yet still smaller) to the ones observed in Croatia (26 %) by Kruzicevic et al. (2012) .

Our results revealed that the majority of students entering medical school have parents with tertiary education and almost one fifth have at least one parent with a medical degree. In the light of statistics showing that within the students’ parents age group (40–50 years old) the percentage of Polish people with tertiary education is around 10 %, it could be anticipated that parental education is one of the factors determining medical career choice. Our observation is in line with the conclusions of a Brazilian study (Millan et al. 2005) that parental higher education and having doctors in the family are facilitators for entering the medical profession.

The motivation for choosing a medical career remained relatively stable throughout the 6 years of study and was a mixture of altruistic and intellectual reasons. Two of the most prevalent factors for choosing medical studies were ‘the desire to help others’ and ‘an interest in medical subjects and issues’. Our observation is consistent with results of studies from other countries (Draper and Louw 2007; Girasek et al. 2011; Millan et al. 2005; Puljak et al. 2007; Todisco et al. 1995). Furthermore, our data showed, that the effect of gender on altruistic motivation was high in the final year students. For male Year 6 students, altruistic motivation moved from first place, occupied at the beginning of the study, to second place, with ‘an interest in medical subjects and issues’ moving from second to first place. Puljak et al. (2007) reported a drop in altruistic motivation among final year medical students but the role of gender was not explained. It could be assumed that the above findings imply that Polish medical students perceived interest in medical knowledge and the desire to care for and help others are at the core of the medical profession. Thus, almost half of the graduating students, irrespectively of gender, considered future involvement in research work.

The leading position of humanitarian and scientific reasons for choosing medical studies corresponds well with the students’ expectations concerning their future medical practice. From among five different expectations related to certainty of finding work, having high prestige and income, further career development, they assigned the greatest importance to ‘the prospect of performing socially important and interesting work’. Among the final year students, the latter expectation was rated significantly higher by women than men (Cohen’s d = 0.472). This may suggest that women are more socially oriented and persistent in maintaining humanitarian and altruistic attitudes throughout the course of medical education. The predominance of female-students in preserving these attitudes at various stages of medical education was also reported in other studies (Wierenga et al. 2003; Heiligers 2012; Dastjerdi et al. 2012).

The most favored specializations, at both points of our survey, were those associated with non-PC career paths and with work in hospital settings. Only a minority of students associated their future career plans with PC specializations. It is worth mentioning that the number of graduates declaring interest in PC specializations almost doubled as compared with freshmen. The increase seemed to be mostly due to a decrease in the number of undecided graduate students. The least popular among the PC specializations was family medicine, as only two Year 1 students (a man and a woman) and two women in Year 6 showed interest in that specialization. A reason for the low interest of Polish students in family medicine and other primary care specializations might be late and limited exposure to these issues during the course of study. Curricula of most Polish medical schools place the ‘family medicine’ module in the final year and the number of hours devoted to this subject varies between 75 and 105, of which seminars/lectures constitute 1/3 and outpatient practice 2/3.

Our data confirmed that Polish students have a positive perception of non-PC career and hospital medicine specializations. That aspect was also highlighted by Pawełczyk et al. (2007) and Krajewski-Siuda et al. (2012). Although, it could be argued that the specialization preferences revealed in the study may change after entering practice, our results point to the substantial disparity between the level of students interest in PC specializations and health care system’s demand for generalist physicians and family doctors. Such a situation requires intensified educational efforts of medical schools and policy makers to make work in the PC sector more attractive. Similar conclusions were drawn from studies carried out on German (Gibis et al. 2012), Hungarian (Girasek et al. 2011), Norwegian (Aasland et al. 2008), Swiss (Buddeberg-Fischer et al. 2006), Turkish (Ozcakir et al. 2007), and US (Compton et al. 2008; Lakhan and Laird 2009) samples. The researchers agree that the motives behind choice of specialty are a combination of professional interests and ambitions, perceived social prestige and position on the one hand and expected lifestyle and family demands on the other. Thus, attempts at promoting work in the PC sector should aim at improving the image of a PC career as well as at creating work conditions that provide a balance between professional activities and personal and family life.

Our data showed a probable link between place of origin and future career plans. Growing up in a town or village was the strongest independent predictor for choosing a PC career. Whereas, those who grew up in larger cities were more likely to choose non-PC specializations. This is in line with other surveys showing that students graduating from rural high schools are more likely to choose PC careers (Pretorius et al. 2008; Rabinowitz et al. 2012). Somewhat weaker independent predictors of PC career preferences were being a female student or a graduating student, and attaching low importance to high earnings. The latter predictor of PC career choice was also noted by Newton et al. (1998) and Knox et al. (2008).

The medical school ethos and curriculum are another variables that seem to affect future career plans connected with specialty choices (Avery et al. 2012). The curricula in Polish medical universities are mostly traditional ones, based on lectures, seminars and laboratories ending with examinations. The 6-years curriculum is divided into 2.5-years of basic science modules and 3.5-years of clinical training with little formal integration between the two parts. Clinical subjects, which start from the 3rd year, are usually held in hospital settings and are devoted to clinical medicine in many subspecialties, often not integrated enough with social and epidemiological aspects (Tatoń and Czech 2008). Such a curriculum overshadows primary care medicine and seems to promote early interest in non-primary care specialties.

The high percentage of final year students (20.5 %) who did not choose a specialty might be connected with the specific situation of Polish graduates. It may be relevant that in Poland a final decision on specialty choice is made after completing a 13-months internship and passing the physicians final examination (LEK), thus a graduating student still has more than a year time to make a decision. The results of LEK determine whether, and to which, residency pathway a person can be accepted. The number of residencies for a particular specialty is established each year by the Ministry of Health and most non-PC specialties are perceived as more attractive than PC specialties. Thus, both the medical curriculum and the results of LEK exam seem to promote choosing non-PC specialties.

To put our results regarding students preferences for future work setting and work sector in a socio-cultural context, it is worth mentioning that until the successful transition of Poland to democracy in 1989 the healthcare system was dominated by the public sector fully funded by the government. All hospitals were state owned, whereas the private sector was limited to single doctor’s private medical practices. Nowadays, all Poles have to contribute to the state healthcare insurance scheme. The state fund covers most medical services in the state owned outpatient clinics and hospitals. The state also contracts some services in privately owned health care institutions. Various types of private medical insurance are also available. There are many specialized private practices and hospitals offering health care. They are created and funded by private operators and through private insurance contributions. Our results show that students seem to have clear preferences regarding their future work setting, from the beginning of their studies. These preferences are further reinforced in the course of their studies. Half of the freshmen and the majority of graduates anticipated working in hospital settings in the future. However, preferences concerning the sector of work appear to be formed later in the course of study. In this regard, more than 40 % of freshmen stated that they didn’t know their preferences and from among those who knew them, the majority preferred work in the private sector. By contrast, all responding Year 6 students specified their preferences and the majority anticipated work either in public or combined public and private sectors. The preference shift, between Year 1 and Year 6, towards favoring work in the public sector seems to correspond with the greater attention paid by Year 6 students to ‘the certainty of finding work’. The fivefold increase in the percentage of students, between Year 1 and Year 6, who wanted to combine work in private and public sector may be considered as an indication that graduating students are sincerely concerned about finding a secure employment option. In this regard, results of other surveys were inconsistent, as students in Germany preferred work in the public over the private sector (Gibis et al. 2012), more students in South Africa opted for the private sector (de Vries et al. 2010), while the majority of students from Angola, Mozambique and Guinea-Bissau preferred to combine work in private and public sectors (Ferrinho et al. 2011).

The strongest predictor of expressed preference for work in the public sector was ‘the sixth year of study’. Other predictors involved lower expectations concerning the size of wages and ‘certainty of finding work’, and a greater desire to perform ‘socially important and interesting work’. This may indicate that students showing preference for future work in the public sector are somewhat more socially minded.

Our results revealed changes in students’ emigration plans between the first and the final year of their studies. A significant decline in the level of interest in seeking employment abroad was observed in the graduating students. Furthermore, being a final year student proved to be the strongest predictor of plans to pursue a future medical career in Poland. Such a tendency is in line with results of recent Polish (Krajewski-Siuda et al. 2012), Hungarian (Girasek et al. 2011), and Swedish (Diderichsen et al. 2011) studies. In the latter investigation three-fold more Year 1 than Year 6 students stated: ‘the world is my workplace’. Thus, it was concluded that freshmen have a more idealistic approach to emigration. The graduating students perspective on work migration might be tempered by the fact that a number of them have already changed marital status and started their own families thus gaining a more realistic approach to life in general.

Other factors that might influence students’ preferences for the sector of future work and work migration plans could be connected with the transition of medical training in Poland to internship and residency. To obtain a license to practice medicine graduates have to complete 13 months of internship in hospitals on a contractual basis and pass the LEK. Results of LEK decide about the prospect of residency training towards a chosen specialization. The residency training usually takes place in accredited public sector units. This may partially explain an increased interest in public sector work among final year students. It seems that the prospect of certain stability during the internship and residency can also play some role in the change of work migration plans among final year students.

Finally, it might be worth considering several limitations of this study. Firstly, our respondents did not comprise a representative sample, as the students came from only one medical university in Poland. However, as all Polish medical schools have similar curricula and receive a comparable level of government funding, the teaching and learning conditions seem to be alike. Thus, it could be assumed that the views and choices of our respondents do not deviate too much from those that are representative of the general population of Polish medical students. Furthermore, the convergence of our results regarding migration plans with those obtained from a cross-sectional survey among Polish medical students recruited from five medical universities (Krajewski-Siuda et al. 2012) offers further support for the above assumption. Secondly, information regarding marital status and family situation of respondents as well as changes that occurred in that respect during a period of five years were not controlled in the study. This meant a loss of control over significant factors influencing career choices (Buddeberg-Fischer et al. 2006; Diderichsen et al. 2011; Heiligers 2012). Thirdly, to ensure full anonymity to our respondents, we did not collect demographic data allowing to match the same student responses at two points of data collection, at the end of Year 1 and at the end of Year 6. Hence, we lost the opportunity to analyze changes on the individual level with paired tests and had to limit our analyses to a group. Another possible limitation of this study might be a bias of social desirability. However, as the survey was anonymous and students were asked to provide honest and sincere responses, we can expect that the potential for the bias of social desirability was rather small.

## Conclusions

Our empirical data, concerning the rather understudied population of Polish medical students, might provide useful information to international readers especially in light of the fact that graduates of Polish medical universities are increasingly gaining employment in other countries of the European Union and beyond. Interestingly, in spite of socio-cultural differences and different educational traditions, the motivation for career choices as well as professional plans and expectations of Polish medical students are, at their core, similar to those reported from various international samples of medical students.

It is worth noting, that our study concentrated on students’ declarations about their future career plans. Thus, it would be worthwhile for future investigations to collect data on the actual decisions those graduates took. An exploration of factors behind the decision making process will allow for a better understanding of what drives the choices made.
